# Development of a High-Performance Liquid Chromatographic Method for Determination of Letrozole in Wistar Rat Serum and its Application in Pharmacokinetic Studies

**DOI:** 10.3797/scipharm.1206-06

**Published:** 2012-08-31

**Authors:** Sasmita Kumari Acharjya, Subrat Kumar Bhattamisra, Bhanoji Rao E. Muddana, Ravikumar V. V. Bera, Pinakini Panda, Bibhu Prasad Panda, Gitanjali Mishra

**Affiliations:** 1 Department of Pharmaceutical Analysis and Quality Assurance, Roland Institute of Pharmaceutical Sciences, Berhampur, Odisha, 760010, India.; 2 Department of Pharmacology, Roland Institute of Pharmaceutical Sciences, Berhampur, Odisha, 760010, India.; 3 Department of Pharmaceutics, Roland Institute of Pharmaceutical Sciences, Berhampur, Odisha, 760010, India.; 4 Department of Zoology; Berhampur University, Berhampur, Odisha, 760007, India.

**Keywords:** RP-HPLC, Letrozole, Rat serum, Pharmacokinetic study

## Abstract

A fast, sensitive, and specific reversed-phase high-performance liquid chromatographic (RP–HPLC) method for the determination of letrozole in Wistar rat serum was developed. In this method, liquid–liquid extraction of letrozole was achieved using diethyl ether as the extracting solvent. The analysis was carried out on a reversed-phase C18 (250 mm × 4.6 mm, 5 μm) column with an isocratic mobile phase of methanol–water (70:30,v/v), at a flow rate of 1.0 mL min^−1^. Detection was carried out at 239 nm with a UV–visible spectrophoto-metric detector. The method was shown to be selective and linear over the concentration range of 0.15–100 μg mL^−1^. The intra-day and inter-day precision studies showed good reproducibility with coefficients of variation less than 11% for the analyte. The relative errors of intra– and inter–day accuracy were within −11.52 to −2.26%. The limit of quantification was evaluated to be 0.15 μg mL^−1^. The method was successfully applied for the pharmacokinetic study of letrozole after oral administration of 10 mg kg^−1^ of letrozole in six healthy Wistar rats.

## Introduction

Letrozole, 4,4′-(1*H*-1,2,4-triazol-1-ylmethanediyl)dibenzonitrile, is a potent, specific, nonsteroidal, third-generation aromatase inhibitor, used therapeutically for the treatment of hormonally responsive breast cancer after surgery [1]. Estrogens stimulate and maintain the cancerous growth of the breast. Treatment of hormonally responsive breast cancers includes a variety of efforts to decrease estrogen levels or inhibit the effects of estrogen. Letrozole prevents the aromatase from producing estrogens by competitive, reversible binding to the heme of its cytochrome P450 subunit. The action is specific, and letrozole does not reduce production of mineralocorticoids or corticosteroids [2]. Letrozole is readily and completely absorbed from the gastrointestinal tract. It is slowly metabolized in the liver to an inactive carbinol metabolite, which is then excreted as the glucoronide in the urine [3].

Various analytical methods for the estimation of the letrozole either alone or in combination with other drugs have been found in the literature such as the UV-spectrophotometric method [4–7], high performance liquid chromatographic method (HPLC) [8–16], microarray approach [17], capillary gas chromatographic method [18], gas chromatography–mass spectrometric method (GC/MS) [19], and the liquid chromatography–tandem mass spectrometric method (LC/MS/MS) [20–22].

The reported UV-spectrophotometric methods [4–7] are not sensitive enough to estimate low concentrations of letrozole in biological fluids. Some of the reported HPLC methods [8, 10–12, 14, 15] have used acetonitrile as one of the mobile phase components which is expensive, toxic, and odorous as compared to methanol. Again, few reported HPLC methods [8, 9] have more than 9 min for the elution time of letrozole, which is not suitable in all conditions, and in few other cases [10, 14] the linearity range is insufficiently sensitive to determine the concentration of letrozole in biological fluids. Pfister et al. [13] have reported the enzyme immunoassay (EIA) and HPLC for determining letrozole in biological fluids, but the HPLC method was not sensitive enough to measure the unchanged compound after administration at low doses (0.5 mg). Although EIA is more sensitive than the HPLC method (0.199 versus 7.988 ng mL^−1^, respectively), its application in clinical samples revealed that it was not specific enough. A strong cross-reactivity of the antibodies with the metabolite has been observed with all urine samples and several plasma samples from patients under repeated administration. Annapurna et al. [16] have reported a HPLC method in which methanol: tetra butyl ammonium hydrogen sulfate was used as the mobile phase (80:20V/V), but the mobile phase containing tetrabutyl ammonium hydrogen sulfate shortened column life. Berzas et al. [18] have developed a capillary gas chromatographic method for the determination of the drugs used in advanced breast cancer; however, thermal decomposition of the drug under GC conditions was the major problem. LC methods based on MS–MS as the detection system for the analysis of letrozole in plasma are sensitive with high selectivity and low quantitation limits, but this procedure is expensive, the analytical method requires highly trained personnel and equipment which is not readily available in most laboratories [20–22].

With this background information, the aim of the present study was to develop and validate a simple, economic, and sensitive HPLC method which has advantages over the reported methods. The developed method has been applied to study the pharmacokinetic profile of letrozole after a single oral administration to Wistar rats. The results of the analysis were validated according to USFDA guidelines [23].

## Experimental

### Chemicals and reagents

The letrozole working standard (purity 99.83%w/w) was obtained as a gift sample from Alembic Ltd., Vadodara, India and further identified by obtaining its melting point, ultraviolet (UV), and infrared (IR) spectra. HPLC grade methanol was purchased from Merck, India. Diethyl ether (low boiling point) was purchased from Finar Reagents, India. High-purity water was prepared using the TKA–GenPure (Germany) water purification system. Solvents were filtered through a 0.45 μm nylon membrane filter and degassed by using an ultrasonicator (Enertech, India). All solutions were prepared daily.

### Instruments and chromatographic conditions

The chromatographic analysis was performed under ambient conditions with the Shimadzu (Japan) HPLC system, which consisted of two pumps (LC–10AT and LC–10AT VP), a system controller (SCL 10AVP), a variable wavelength programmable UV–VIS spectrophotometric detector operated at 239 nm (SPD–10A), and a manual injection valve with a 20 μL filling loop. The output signal was monitored and integrated using Shimadzu CLASS-VP V 6.14 SP1 software. The analysis was performed on a reverse-phase column (250 mm × 4.6 mm i.d.) which was packed with 5 μm particles of Hypersil ODS C18 packing material (Hibar^®^ LiChrospher^®^). The mixture of methanol–water was used as the mobile phase (70:30, v/v) at a flow rate of 1.0 mL min^−1^. The eluent was filtered through a 0.45 μm nylon membrane filter and degassed by using an ultrasonicator.

### Preparation of stock and standard solutions

Stock solution of letrozole (8000 μg mL^−1^) was prepared by dissolving the drug in methanol and further diluted with methanol to obtain the different working standard solutions ranging from 6–4000 μg mL^−1^. All of the solutions were refrigerated (4°C) when not in use and were stable for two weeks.

### Preparation of calibration standards

Working standard solutions (50 μL) within the concentration range of 6–4000 μg mL^−1^ was pipetted into screw cap tubes containing 200 μL of the blank serum. Then, 500 μL of diethyl ether was added and vortexed to mix for 2 min which was further centrifuged (5 min at 6000 × *g*). The supernatant diethyl ether layer (250 μL) was separated and evaporated at 40°C using a water bath. The evaporated residue was reconstituted with 1 mL of the mobile phase to get concentrations of 0.15–100 μg mL^−1^ of the drug. The calibration curve of letrozole in serum was prepared by injecting 20 μL of the syringe-filtered (0.45 μm syringe filter), reconstituted standard solution.

### Preparation of the sample solutions

The aliquot (250 μL) of the Wistar rat serum containing letrozole was pipetted into screw cap tubes. To this, 500 μL of diethyl ether was added and vortexed for 2 min which was further centrifuged (5 min at 6000 × *g*). The supernatant diethyl ether layer (250 μL) was separated and evaporated at 40°C using a water bath. The evaporated residue was reconstituted with 1 mL of the mobile phase. 20 μL of the syringe-filtered (0.45 μm syringe filter), reconstituted sample solution was injected into the HPLC system.

### Method validation

The method was validated according to the guidelines of USFDA with respect to specificity, accuracy, precision, recovery, linearity, and stability of analyte in spiked samples [23].

#### Specificity

To determine the specificity of the method, blank Wistar rat serum, serum spiked with known amounts of the drug (10 μg mL^−1^), and serum samples from Wistar rats after the oral dose of drug were analyzed.

#### Linearity

The linearity was evaluated over the concentration range of 0.15–100 μg mL^−1^ at seven levels (0.15, 0.5, 5, 10, 20, 50, and 100 μg mL^−1^). The calibration curve for letrozole (unweighted regression line) was obtained by the linear least-squares regression analysis by plotting peak–area (*y*) versus the theoretical concentrations of standards (*x*).

#### Limit of detection and limit of quantification

The limit of detection (LOD) is defined as the concentration of analyte giving a signal-to noise-ratio of 3:1. The lower limit of quantification (LLOQ) is defined as the lowest concentration of letrozole in the calibration curve, giving an acceptable accuracy (RE) within ± 20% and a precision (RSD) that did not exceed 20%.

#### Extraction recovery

The extraction recovery of letrozole from serum, at the concentration range of the calibration curve, was determined at the concentrations 0.5, 20.0, and 50.0 μg mL^−1^. Five replicates of each concentration were prepared by the above-mentioned sample preparation technique and injected into the HPLC system. The extraction recovery at each concentration was calculated using the following equation:

% Recovery=(a/b)×100

Where, a = Peak area obtained after extraction of known amounts of letrozole from serum and b = Peak area obtained from the same amounts of unextracted letrozole

#### Precision and accuracy

The intra- and inter-day precision and accuracy of the assay were determined by percent relative standard deviation (RSD) and percent relative error (RE) values, respectively. The intra- and inter-assay precisions were evaluated by analyzing the samples at three concentration levels of letrozole (0.5, 20.0, and 50.0 μg mL^−1^). For the intra-day validation, five replicates of the serum samples were analyzed on the same day. For the inter-day validation, five replicates of the serum samples were analyzed on three different days. The precision was expressed as the %RSD which should not exceed 15%, except at the LLOQ where it should not exceed 20%. The accuracy of the assay was determined by comparing the means of the determined letrozole concentrations with the nominal concentrations. The mean percentage deviation from the nominal values were expressed as the %RE, which should be within ± 15% of the nominal value, except at the lower limit of quantification where it should not exceed ± 20%.

The % RE values were calculated by the following equation:

Percent relative error=[(x-y)/y]×100

Where, *x* = Calculated concentration; and *y* = Added concentration

#### Stability

To ensure the reliability of the results in relation to the handling and storing of serum samples and stock standard solutions, stability studies were carried out at two different concentration levels, 0.5 and 20.0 μg mL^−1^. The freeze-thaw stability was tested after three freeze (24 h storage, −20°C) and thaw (room temperature for 2–3 h) cycles, and the long-term stability was studied by storing samples at −20°C for 2 weeks. The post-preparation stability was assessed by analyzing the samples after 12 h of storing at 25°C.

### Animals and pharmacokinetic study

The pharmacokinetic study was conducted in six male Wistar rats (body weight 200–250 g), with the permission from the Institutional Animal Ethics Committee (IAEC), Roland Institute of Pharmaceutical Sciences, Berhampur, India. Before starting the experiment, Wistar rats were kept in an environmentally controlled room for one week and fed with standard laboratory food and water *ad libitum*. The rats fasted overnight before the experiment. Rats were given the letrozole suspension (prepared by using 1% sodium carboxy methyl cellulose) orally at doses of 10 mgkg^−1^ body weight. Blood samples (0.5 mL) were collected sublingually at the intervals of 0.0, 0.5, 1.0, 2.0, 4.0, 6.0, 12.0, 24.0, 48.0, and 72.0 h. All blood samples were allowed to clot and were centrifuged (ELTEK RC 4815 F, India) for 10 min at 3000 g. The serum was separated and transferred into clean microcentrifuge tubes and stored at −20°C until HPLC analysis. Physiological saline (0.5 mL) was administered to compensate for the blood loss after each blood draw. As per the single compartmental pharmacokinetic model, different pharmacokinetic parameters like peak serum concentration (*C*_Max_), time to reach peak concentration (*T*_Max_), area under the curve (AUC_0-t_ and AUC_0-∞_), half life (*t*_1/2_), elimination rate constant (K), and volume distribution (V_d_) were calculated by using the software PK functions for Microsoft Excel (Joel Usanky, Atul Desai, and Diane Tang-Liu). Further, these plasma concentration *vs* time profile data were exposed to the noncompartmental model (statistical moment theory), and then the area under the moment curve (AUMC_0-t_ and AUMC_0-∞_), and mean residence time (MRT) were determined.

## Results and discussion

### Chromatography and extraction procedure

**Fig. 1.**
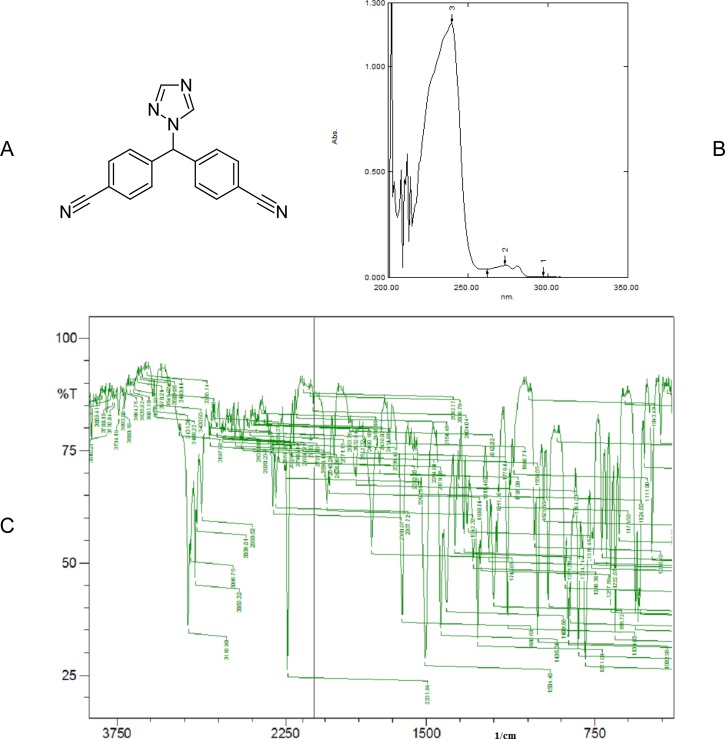
(A) Chemical structure of letrozole (CAS number 112809-51-5) (B) UV absorption spectrum of letrozole between 200 and 350 nm (C) IR spectrum of letrozole

Identity of the standard drug letrozole (Fig. 1a) was evaluated by obtaining its melting point (184.6°C), and recording its UV absorption spectra (Fig. 1b) and infrared spectra (Fig. 1c). To carry out the HPLC analysis, initially various mobile phase compositions were attempted in order to obtain a rapid and simple assay method for the determination of letrozole. Then the mixture of methanol and water (70:30, v/v) at a flow rate of 1.0 mL min^−1^ was selected as the mobile phase based on peak parameters (asymmetric factor and theoretical plates were 1.03 and 3086.19, respectively), retention time (3.58 ± 0.009 min), ease of preparation, and cost. The detection wavelength was set at 239 nm, because at this wavelength letrozole shows maximum absorbance in the UV absorption spectrum. The analytical column was equilibrated using the eluting solvent system, and by maintaining the optimized chromatographic conditions. After an acceptable stable baseline was achieved, the standards and samples were analyzed. For serum sample preparation, the liquid–liquid extraction technique was used because in the protein precipitation technique, interference was observed and also extraction efficiency was lower.

### Method validation

#### Specificity

**Fig. 2.**
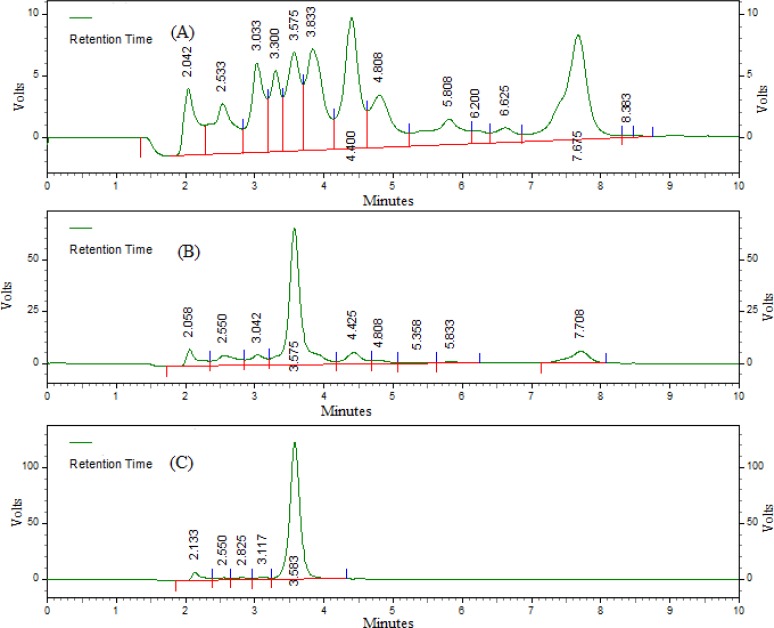
Representative HPLC chromatograms of (A) blank rat serum, (B) serum spiked with 10 μg mL^−1^ letrozole, and (C) serum samples at 2 h after an oral administration of letrozole at a dose of 10 mgkg ^−1^ body weight.

Typical chromatograms of blank serum, spiked serum, and serum sample at 2.0 h after an oral administration of letrozole at a dose of 10 mgkg^−1^ body weight are given in Fig. 2. This shows no interfering peaks in the region of the location of the peak of the analyte. The retention time of letrozole was 3.58 ± 0.009 min and the total run time was 10 min. The method was equally specific as that of the reported method [9].

#### Linearity

The evaluation of the linearity was performed with a seven-point calibration curve over the concentration range of 0.15–100 μg mL^−1^. The slope and intercept of the calibration graph was calculated by using linear regression analysis. The regression equation of the calibration curve was: *y* = 71726 *x* – 37558; *r =* 0.998, where *y* is the peak area of letrozole, and *x* is the serum concentration of letrozole. The linearity range of the developed method was more when compared to the reported method, where the linearity range was 0.0005–0.080 μg mL^−1^[10] and 0.05–0.12 μg mL^−1^[11].

#### Limit of detection and limit of quantification

The limit of detection was 0.045 μg mL^−1^at a signal-to-noise ratio of 3:1, and the lower limit of quantification was 0.15 μg mL^−1^, with the precision and accuracy within 15% verified by repeated analysis. The method is almost equally sensitive as that of the reported method [11], where the limit of quantification was 0.0375 μg mL^−1^and the limit of detection was 0.0125 μg mL^−1^ for letrozole.

#### Extraction recovery

**Fig. 3.**
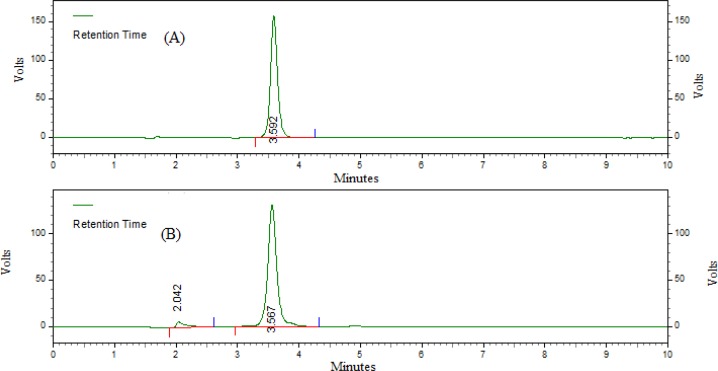
Representative HPLC chromatograms of (A) unextracted letrozole and (B) extracted letrozole from rat serum.

The extraction recoveries of letrozole from serum at three concentrations (0.5, 20.0, and 50.0 μg mL^−1^) are shown in Tab. 1 and in Fig. 3. The average recovery of letrozole was 96.94 ± 2.66%, which suggested that there was negligible loss during extraction.

#### Precision and accuracy

The accuracy and precision of the method were evaluated with samples at three concentrations and using five replicates. The results are shown in Tab. 2. The intra- and inter-day precisions were satisfactory with the RSD less than 10.14 and 10.69%, respectively. The RE of intra- and inter-day accuracy were within −11.52 to −2.26%. The precision and accuracy study indicated that the developed HPLC method was reproducible and accurate.

#### Stability

The results of the stability study involving samples at two levels (0.5 and 20 μg mL^−1^, *n* = 5) are presented in Tab. 3. The RSD was below 10.26% and the RE was within −11.56 to −2.91%, which confirmed the high stability of letrozole throughout the determination.

### Pharmacokinetic studies

**Fig. 4.**
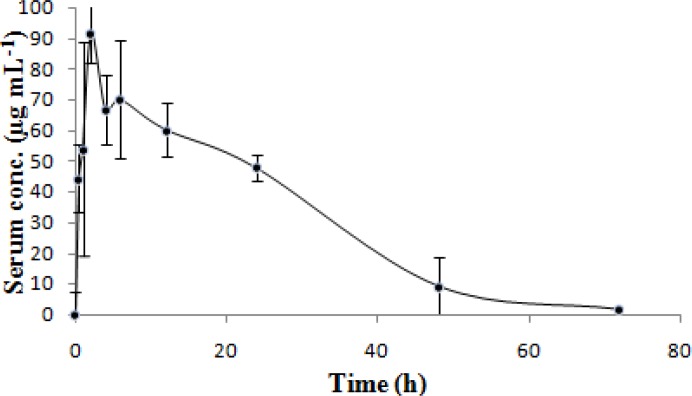
Mean serum concentration–time profile of Letrozole in Wistar Rat after oral administration (n= 6, dose = 10 mg kg^−1^). Values were expressed as mean ± SD.

Pharmacokinetic data were calculated by using both the compartmental and noncompartmental models. The serum concentration–time curve of letrozole in Wistar rats following oral administration of 10 mgkg^−1^ body weight are shown in Fig. 4 and the derived pharmacokinetic parameters are summarized in Tab. 4. These pharmacokinetic parameters are in good agreement with those found previously, and no significant difference was observed between our pharmacokinetic data and results reported in the literature [24].

## Conclusion

A simple and specific HPLC method for the determination of letrozole in Wistar rat serum was developed and successively applied to an *in vivo* kinetic study in rats. Using this method, it allowed us to investigate the pharmacokinetics of letrozole in rats after oral administration of letrozole at a dose of 10 mg kg^−1^ body weight. This investigation contributes not only to the determination letrozole in rat serum by HPLC, but also to our understanding of the linear pharmacokinetic characteristics of letrozole over the dose studied in rats after oral administration.

## Figures and Tables

**Tab. 1.** Recovery of Letrozole from rat serum

**Added concentr. (μg mL^−1^)**	**Mean detected concentr. (μg mL^−1^)**	**Recovery (%)**	**RSD (%)**
0.5	0.49 ± 0.01	96.76 ± 2.01	2.08
20	19.52 ± 0.76	97.61 ± 3.78	3.87
50	48.22 ± 1.10	96.44 ± 2.20	2.28
Average		96.94 ± 2.66	2.75

n = 5; Values were expressed as mean ± SD.

**Tab. 2.** Precision and accuracy of Letrozole in rat serum

**Spiked concentr. (μg mL^−1^)**	**Intra-day**			**Inter-day**		

	**Mean detected concentr. (μg mL^−1^)**	**RSD (%)**	**RE (%)**	**Mean detected concentr. (μg mL^−1^)**	**RSD (%)**	**RE (%)**
0.5	0.45 ± 0.05	10.14	–10.36	0.44 ± 0.05	10.69	–11.52
20	19.22 ± 0.49	2.53	–3.89	19.22 ± 0.53	2.77	–3.89
50	49.07 ± 0.87	1.77	–1.89	48.87 ± 0.82	1.68	–2.26

Intra-day: n=5; Inter-day: n=3 days with 5 replicates per day; Values were expressed as mean ± SD.

**Tab. 3.** Stability of Letrozole in rat serum

**Stability**	**Spiked concentr. (μg mL^−1^)**	**Mean detected concentr. (μg mL^−1^)**	**RSD (%)**	**RE (%)**
Freeze-thaw^a^	0.5	0.46 ± 0.03	6.1	–8
Freeze-thaw^a^	20	19.42 ± 0.32	1.7	–2.9
Long-term^b^	0.5	0.44 ± 0.04	9.6	–11.6
Long-term^b^	20	19.37 ± 0.57	2.9	–3.2
Post-preparative^c^	0.5	0.45 ± 0.05	10.3	–10.3
Post-preparative^c^	20	19.22 ± 0.58	3.0	–3.9

a After three Freeze (−20°C) and Thaw (room temperature) cycles;

b After two weeks of storing at −20°C;

c After 12 h of storing at 25°C; Values are expressed as mean ± SD (n= 5).

**Tab. 4.** Mean Pharmacokinetic parameters of Letrozole in Wistar Rat (n= 6) after oral administration of 10 mg Kg^−1^ body weight

**Parameters**	**Compartmental Model**	**Non-Compartmental Model**
C_max_ (μg mL^−1^)	88.82	–
T_max_ (h)	8.9	–
K (h^−1^)	0.06	0.06
*t*_1/2_(h)	10.76	–
AUC_0–t_ (μg h mL^−1^)	2259.95	–
AUC_0–∞_ (μg h mL^−1^)	2287.58	–
Volume of distribution (L)	0.0127	–
AUMC_0_t_ (μg h^2^ mL^−1^)	–	41904.11
AUMC_0–∞_ (μg h^2^ mL^−1^)	–	44913.53
MRT (h)	–	19.6

C_max_= peak serum concentration; T_max_= time to reach peak concentration; K= Elimination rate constant; *t*_1/2_= Half-life; AUC_0–t_= area under the curve from zero to t; AUC_0–∞_= area under the curve from zero to infinite; L= Volume of distribution; AUMC_0_t_= area under the moment curve from zero to t; AUMC_0–∞_= area under the moment curve from zero to infinite; MRT= mean residence time.
